# The Global Expansion of Quinoa: Trends and Limits

**DOI:** 10.3389/fpls.2016.00622

**Published:** 2016-05-09

**Authors:** Didier Bazile, Sven-Erik Jacobsen, Alexis Verniau

**Affiliations:** ^1^UPR Green, Department of Environment and Societies, French Agricultural Research and International Cooperation Organization, CIRAD, MontpellierFrance; ^2^Department of Plant and Environmental Sciences, Faculty of Science, University of Copenhagen, TaastrupDenmark; ^3^Ecole Supérieure d’Agricultures, AngersFrance

**Keywords:** *Chenopodium quinoa* Willd., plant genetic resources, adaptation, climate change, benefit-sharing seed regulations, farmers’ rights

## Abstract

Quinoa (*Chenopodium quinoa* Willd.) was first domesticated in Andean countries over 7000 years ago. Following the Spanish conquest, quinoa was rejected as “Indian food.” After centuries of neglect, the potential of quinoa was rediscovered during the second half of the 20th century. Since then, the number of countries importing quinoa increased, with new producers appearing on the map and quinoa now being cultivated in areas outside the Andean countries. The geographical increase in distribution of quinoa has highlighted the difficulty of access to quality seed, which is a key factor for testing the crop outside the Andes. In this context, research partnerships have helped promote the exchange of quinoa germplasm and have allowed trials to be undertaken in non-traditional areas of cultivation. The number of countries growing the crop has increased rapidly from eight in 1980, to 40 in 2010, and to 75 in 2014. A further 20 countries have sown quinoa for the first time in 2015. In this paper, we analyze this trend and discuss the limits of quinoa’s expansion. As commercial production of quinoa is expected to develop, changes in international regulatory frameworks on genetic resources are needed in order to facilitate plant breeding for the most adaptive varieties for each region.

## Introduction

Quinoa (*Chenopodium quinoa* Willd.), is an annual species that originates from South America. Its domestication is thought to have begun in Andean region around 7000 years ago. Generations of farmers have been involved in quinoa selection, which explains the high levels of genetic diversity found today.

Quinoa has remained a staple food for indigenous people of the Andes over the centuries. Following the Spanish conquest, quinoa was rejected as “Indian food” but it has never disappeared despite the introduction of Old World species. After centuries of neglect, the nutritional status of quinoa was rediscovered during the second half of the 20th century, leading to a renaissance of its production (Repo-Carrasco et al., 2003).

Thanks to the high levels of genetic diversity, the crop is highly resilient to agro-ecological extremes (soils, rainfall, temperature, and altitude) and is tolerant to frost, drought, and salinity (Ruiz et al., 2014, 2015). Quinoa can be divided into different groups or ecotypes, reflecting its diffusion from the center of origin around Lake Titicaca. Each of these ecotypes is associated with sub-centers of diversity (Risi and Galwey, 1984), and highly adapted to specific environments. For example, the sea level ecotype from the central and southern part of Chile is the most adapted to temperate environments (Galwey, 1993; Jacobsen, 1997, 2003; Christiansen et al., 2010; Bendevis et al., 2014a,b), useful in developing new varieties for northern latitudes (Jacobsen, 2015). Quinoa diversity is divided into five main ecotypes (Bazile et al., 2013):

• Highlands in Peru and Bolivia;
• Inter-Andean valleys in Colombia, Ecuador, and Peru;
• Salares in Bolivia, Chile, and Argentina;
• Yungas in Bolivia;
• Sea level in Chile.

Research partnerships have often facilitated the exchange of germplasm and have had a powerful impact on this development by strengthened collaborations (Izquierdo et al., 2003). However, partnerships between research institutions for germplasm exchanges need to consider legal and ethical aspects related to the access to genetic resources for experimentation and fair commercial development (Bazile, 2015).

Today, quinoa is still considered a minor crop for global food and agriculture and often classified as a Neglected and Underutilized Species (NUS) with a high potential of development. However, its current distribution is now changing due to changes in consumption patterns. In this article, we analyze this trend, and present a case for the increase in production of quinoa around the world.

## Synthesis of Existing Data

A survey was conducted of resource persons who have been involved in quinoa research over the global level during the last 30 years. The survey identified those countries involved in quinoa research and cultivation. In addition, an analysis was conducted on the issues of access to quality seed of quinoa.

Quinoa project reports were studied to complement the information obtained through surveys. The genotypes used were described, together with their agromorphological characteristics and their origin was determined. The data generated was used to identify the key elements in the expansion of quinoa’s cultivation.

## A Neglected and Underutilized Species (NUS) With a Rapid Expansion

From at least 7000 years ago until the beginning of the 1980s, quinoa has only been connected to the Andes. However, when researchers in other countries understood the potential and benefits of quinoa, experimentation has not stopped growing. Studies have been performed in an increasing number of countries. The number of countries growing quinoa has risen rapidly from 8 in 1980 to 75 in 2014, with a further 20 countries which sowed quinoa for the first time in 2015 (Bazile and Baudron, 2015).

The first known experiment conducted outside the Andes took place in 1935 in Kenya (Elmer, 1942) using the cream-colored seed variety, obtained from the Royal Botanical Gardens, Kew, UK. Trials were conducted the response of quinoa to various nutrient deficiencies in 1948, on the tolerance of quinoa to salinity in 1950, and quinoa’s growth response to temperature in 1968. Research was then undertaken using Chilean germplasm in the 1980s led by Colorado State University, USA. The same time period saw the beginning of commercial quinoa cultivation in Canada. Other countries followed and quinoa was introduced to UK (1983), Denmark (1984), Tibet (1984), India (1985), The Netherlands (1986), China (1988), Brazil and Cuba (1989) (Bazile et al., 2015).

The project “American and European Test of Quinoa” (Mujica et al., 2001) overseen by Angel Mujica and Sven-Erik Jacobsen together with Juan Izquierdo from the Food and Agriculture Organization of the United Nations (FAO) that began in 1996, was an important effort in the 1990s. The testing followed two other important activities, which were focused on cultivation in Europe: the EU project under the AIR^1^ program “Quinoa – a multiple crop for EC’s Agriculture diversification, 1993–1997,” coordinated by University of Cambridge, UK, and the Food and Agriculture COST Action 814 on “Crop Adaptation to Cool and Wet Climates, 1991–1996,” coordinated by the Ministry of Agriculture, Belgium. Through international cooperation initiatives for promoting quinoa, field trials were set up in other countries such as Sweden, Poland, Czech Republic, Austria, Germany, Italy, and Greece.

**FIGURE 1:**
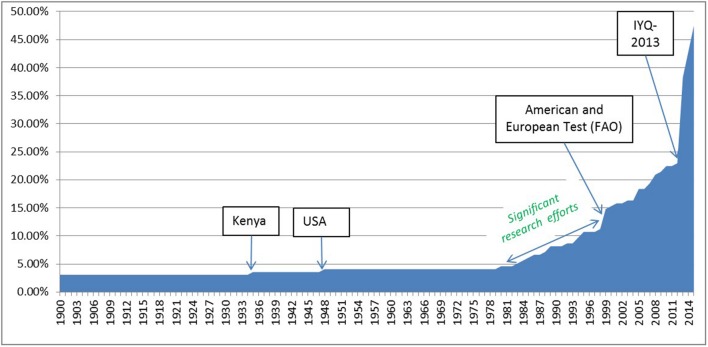
**Percentage of UN countries with quinoa experimentation or cultivation (1900–2015)**.

The General Assembly of the United Nations declared 2013 as the International Year of Quinoa (IYQ). Quinoa was afforded a high profile as a crop with the potential to grow in importance in world agriculture (Jacobsen, 2003; Ruiz et al., 2015). The rapid expansion of the harvested area, with a doubling of countries from 2013, is rapidly changing the perception and representation of quinoa from a minor to a potential major crop (**Figure 1**).

The main quinoa producers in the world, however, are still Bolivia and Peru. In 2013, the area under quinoa cultivation in Bolivia was 75000 ha and in Peru 45000 ha. These two countries produce more than 80% of quinoa in the world, followed by Ecuador, USA, China, Chile, Argentina, France, and Canada, which together represent 15–20% of the world production (Bazile, 2015; Bazile et al., 2015). The area under quinoa cultivation in Europe has increased from 0 in 2008 to 5000 ha in 2015, mainly in France, Spain, and UK (Abbott, personal communication). China has first experimented with cultivating quinoa in Tibet in 1984, while today quinoa is cultivated in nine Chinese regions and over 2500 ha.

The activities carried out due to the IYQ have directly contributed to awareness raising for quinoa cultivation. FAO is actively involved in testing quinoa in 27 countries outside the Andean region, with the aim to promoting food security, due to the nutritional qualities of quinoa and its resistance to abiotic stresses. During 2013–2015, evaluation of quinoa varieties were conducted in:

• Central and Southern Asia (Kyrgyzstan, Tajikistan, Sri Lanka, and Bhutan);
• Western Asia and North Africa (Algeria, Egypt, Iraq, Iran, Lebanon, Mauritania, Sudan, and Yemen);
• Africa (Djibouti, Kenya, Somalia, South Sudan, Ethiopia, Uganda, Zambia, Burkina Faso, Cameroon, Chad, Niger, Senegal, Togo, Ghana, and Guinea).

Size, shape, and compactness of the inflorescence are considered to be important for the rate of maturation. A large open inflorescence will dry quicker after rain and morning dew than a small, compact one, but it may also be prone to seed loss, as quinoa is relatively little domesticated, with few, modern varieties available (Jacobsen, 1997).

The breeding of quinoa, especially Europe and North America, concentrated on gains in time to maturity (around 150 days), high yield and uniformity (Zurita-Silva et al., 2014; Peterson et al., 2015). Another aim has been to reduce the content of saponins in the seed hull. Saponin is a bitter-tasting compound acting as a general defense against biotic stresses. For commercial production, however, the removal of saponins is an additional cost. As a result, sweet cultivars have been developed for use in Europe, especially in the Netherlands (Mastebroek et al., 2000, 2002) and in Denmark (Jacobsen, 2015).

Today quinoa is presently cultivated or tested in 95 countries of the world (Bazile, 2015). This global expansion of quinoa looks set to continue as increasing numbers of countries are testing quinoa.

## Global Regulatory Frameworks for Quinoa Cultivation

Considering the evolution of global regulations on seeds, two periods must be distinguished for their importance on biodiversity sovereignty: the signing of the Convention of Biological Biodiversity (CBD in Rio de Janeiro, Brazil in 1992) marks a particularly break and contrasts the before and the after for plant genetic resources’ access and sharing. The CBD stipulates national sovereignty over biodiversity and obliges parties to form bilateral agreements for accessing their genetic resources (United Nations, 1992). In order to facilitate innovation in plant breeding, the International Treaty on Plant Genetic Resources for Food and Agriculture of 2004 provides a specific instrument for exchanging germplasm of the major food and forage crops (FAO, 2009). It established a global and Multilateral System (MLS) to allow farmers, plant breeders, and scientists to exchange plant genetic materials (Louafi et al., 2013).

Quinoa, unfortunately, is not one of the species pertaining to Annex 1 of the Treaty, which is a list of those species included the MLS of exchanges. The Declaration of Cordoba (2012) from the International Seminar “Crops for the XXI Century,” the first international event celebrating the UN IYQ 2013, proposed the addition of minor crops to be included in the Annex I to the Treaty. To date, however, there has been no consensus reached.

The different regulations on plant genetic resources are usually applied at different levels (local, national, and international) and for different purposes (genetic resources, varieties and seeds, agricultural by-products, etc.). For quinoa, however, there is no single existing legal framework providing a comprehensive coverage of all the issues related to the genetic resources and their sustainable management (Chevarria-Lazo et al., 2015). The CBD does regulate bilateral access and benefit sharing, but this is difficult to apply to quinoa as the crop is now planted internationally, not restricted to the Andean region, and this has been the case for decades.

Prior to 1992, 25 countries had developed *ex situ* collections of quinoa accessions, which are now distributed without legal necessity of prior agreement from Andeans countries (Rojas et al., 2015). As many countries had established collections prior to coming into force of the CBD, this now means that these countries may develop new varieties from this germplasm without having to refer to the country of origin. Nevertheless, individuals and institutions do not have the right to share quinoa germplasm outside their country as per the terms of the CBD, but this pertains only to those countries that have ratified the CBD. A country a signatory to the CBD, however, has only a moral obligation not to go against it. To date, the CBD has been ratified by 196 countries (the Parties), and 168 have signed it before. However, there are a few countries, such as the USA, have not yet ratified this convention.

Overall, these legal regulations or restrictions at the global level to exchange germplasm across countries, only a small part of quinoa’s genetic diversity is available to serve quinoa’s adaptation in new environments for expansion of its cultivation.

**FIGURE 2:**
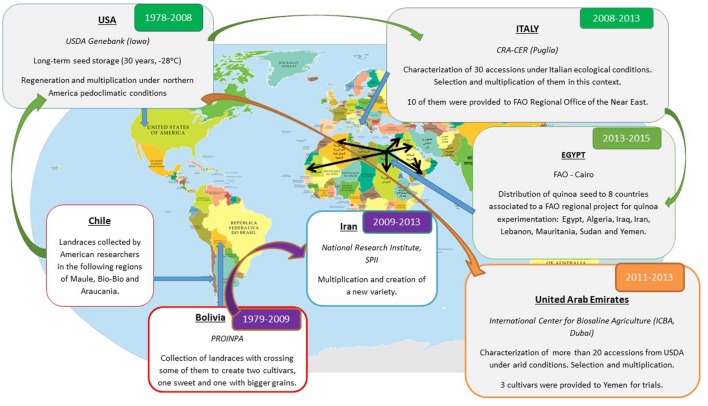
**An example of quinoa seed exchanges from Andean countries to new producers’ countries**.

Exchange of quinoa germplasm has been undertaken both formally via legal provisions (Standard Material Transfer Agreements – SMTAs), or informally via research networks (**Figure 2**). Only 25% of genetic material exchanges correspond to individual exchanges, while 75% of the material has been exchanged among research networks. Considering personal links into existing partnerships, more than 85% of the respondents of the survey declared not signing MTAs.

## Geneflows of Quinoa

Early stages of expansion highlighted the interest among importing countries and consumers for adapting quinoa to new environments, and it facilitated exchange of genetic resources. Now we are entering another phase of quinoa development, and a turning point with many countries interested in quinoa cultivation but with limitations for access to genetic resources.

**Figure 2** illustrates the seed exchange among different partners involved in quinoa trials. Collections of quinoa accessions were created during the 1980s by Wilson (1988a,b, 1990) and taken back to the USA. The United States Department of Agriculture (USDA), Iowa, USA, maintains over 229 accessions, which are sent to those who request them. In Italy, the Cereal Research Centre (CER-CRA) obtained and evaluated 100 accessions for cultivation and development of varieties adapted to Mediterranean conditions. After undertaking germplasm characterization, low performing accessions were eliminated and some of the selected accessions were then distributed to Egypt (**Figure 2**). The FAO Regional Office for Near East in Cairo, Egypt, assisted in distributing quinoa accessions among National Research Institutions from eight countries (Algeria, Egypt, Iraq, Iran, Lebanon, Mauritania, Sudan, and Yemen) to conduct an evaluation of these genotypes in semi-arid and arid conditions. This example of seed flow demonstrates the importance of research networks in distributing quinoa seed at the global level. It also has limitations because of change of codes (unique identifier assigned to the accession in a genebank) from one genebank to another, and from Andean countries to other countries. Avoiding code change is one main challenge for data and germplasm sharing with transparency, but sometimes code change serves as a form of protection for plant breeders to mask the origin of the material they are developing.

The current situation provides a specific legal framework for access and exchange of genetic resources that have a strong impact on use and innovation. The Nagoya Protocol on Access and Benefit-sharing was adopted in Japan in 2010, entering in force in 2014 (70 Ratifications and 92 Signatures). It provides an international agreement, which aims at sharing the benefits from the utilization of genetic resources in a fair and equitable way, and contributing to the conservation of biological diversity and the sustainable use of its components. Considering the limits of the CBD for innovation in plant breeding, the Nagoya Protocol provides a new pathway for enhancing legal transparency on procedures for access and benefit-sharing, and for monitoring the utilization of genetic resources. Andean countries need to be active in this international agenda if they want to protect their interests on quinoa. Solutions need to be developed to better harmonize the different existing legal frameworks and to create new complementary ones. The rapid spread of quinoa at the global level provides an opportunity to consider more in depth the implications on seed exchange of the current regulatory instruments for genetic resources, so that they can be improved and implemented. In addition, it also offers an opportunity for plant breeders to reconsider models of varietal innovations in plant breeding to manage genetic resources for recombination without registration of intellectual property rights (IPR) for new varieties. In addition, adaptation of varieties to global climate changes needs to cope with an increasing unpredictability. Genetic structure of future varieties must be able to evolve to face those changes (Murphy et al., 2016). Landraces (crop populations with high level of intra-varietal diversity) from Central and Southern Chile, and in some cases from the highlands of Peru and Bolivia, are those most frequently often tested outside the Andes. In addition, researchers are mostly using only three varieties (Puno, Regalona, and Titicaca) registered through Plant Variety Protection (PVP; Bendevis et al., 2014a,b; Jacobsen, 2015). The geographical expansion of quinoa cultivation has led to an increase in the number of PVP into action worldwide. Some countries (Netherlands, Chile, Canada, Denmark, Peru, UK, and Israel) have developed new varieties, and have set up a PVP certificate in the UPOV system. These commercial varieties facilitate access to seeds for quinoa testing. However, due to their physiological similarity being less day length sensitive than original material from Peru and Bolivia, the genetic diversity for quinoa experimentation and adaptation in new environments is narrow. Genebanks in the Andean region conserve more than 88% of the accessions available worldwide, which is considerable when taking into account the 16,422 accessions of quinoa and its wild relatives are conserved in 59 genebanks in 30 countries (Rojas et al., 2015). Despite the high levels of quinoa diversity conserved, this is currently not exchanged globally due to highly political issues of national sovereignty (Bazile, 2015).

Intellectual Property Rights should be addressed, recognizing the work of the Andean people in the selection and conservation of local quinoa landraces (Fuentes et al., 2012), and maintaining and adding value to quinoa’s biodiversity for the benefit of world food security and poverty reduction. The future global seed system for quinoa cultivation needs to focus on increasing linkages among farmers, researchers, plant breeders, and politicians. This requires an iterative dialog at the national, regional, and international levels among all the stakeholders involved in managing and using quinoa diversity.

## Author Contributions

DB made the survey and networks analysis on quinoa global expansion and has written the first draft of the manuscript for revision by the co-authors. He is currently developing a Global Collaborative Network on Quinoa. S-EJ participated in many introduction of quinoa around the world. He shared his experience to integrate his global view on quinoa expansion to increase the quality of the mini-review. AV participated in various national projects on quinoa trials in the Near East and North Africa during his studies. He mapped the seed exchanges with the example of (**Figure 2**) in this manuscript.

## Conflict of Interest Statement

The authors declare that the research was conducted in the absence of any commercial or financial relationships that could be construed as a potential conflict of interest. The reviewer EL and handling Editor declared their shared affiliation, and the handling Editor states that the process nevertheless met the standards of a fair and objective review.

## Footnotes

1 FP3-AIR – Specific research and technological development and demonstration programme (EEC) in the field of agriculture and agro-industry, including fisheries, 1990–1994.
